# Iodomethylcholine Inhibits Trimethylamine-N-Oxide Production and Averts Maternal Chronic Kidney Disease-Programmed Offspring Hypertension

**DOI:** 10.3390/ijms24021284

**Published:** 2023-01-09

**Authors:** You-Lin Tain, Guo-Ping Chang-Chien, Sufan Lin, Chih-Yao Hou, Chien-Ning Hsu

**Affiliations:** 1 Department of Pediatrics, Kaohsiung Chang Gung Memorial Hospital, Kaohsiung 833, Taiwan; 2 College of Medicine, Chang Gung University, Taoyuan 330, Taiwan; 3 Institute of Environmental Toxin and Emerging-Contaminant, Cheng Shiu University, Kaohsiung 833, Taiwan; 4 Center for Environmental Toxin and Emerging-Contaminant Research, Cheng Shiu University, Kaohsiung 833, Taiwan; 5 Super Micro Mass Research and Technology Center, Cheng Shiu University, Kaohsiung 833, Taiwan; 6 Department of Seafood Science, National Kaohsiung University of Science and Technology, Kaohsiung 811, Taiwan; 7 Department of Pharmacy, Kaohsiung Chang Gung Memorial Hospital, Kaohsiung 833, Taiwan; 8 School of Pharmacy, Kaohsiung Medical University, Kaohsiung 807, Taiwan

**Keywords:** developmental origins of health and disease (DOHaD), hypertension, chronic kidney disease, trimethylamine-N-oxide, trimethylamine, gut microbiota

## Abstract

Chronic kidney disease (CKD) affects 10% of the global population, including pregnant women. Adverse maternal conditions determine the developmental programming of many diseases later in life. We previously demonstrated that adult rat offspring born to dams with CKD developed hypertension and renal hypertrophy. Trimethylamine-N-oxide (TMAO), a uremic toxin derived from the gut microbiota, has been linked to hypertension. This study assesses the effects of TMAO inhibition by iodomethylcholine (IMC) treatment on offspring hypertension programmed by maternal CKD. Female rats were fed either a control or a 0.5% adenine diet before conception, with or without IMC treatment during pregnancy and lactation. Maternal IMC treatment averted maternal CKD-primed offspring hypertension and renal hypertrophy in 12-week-old offspring. Offspring hypertension is associated with increases in the plasma TMAO concentration and oxidative stress and shifts in gut microbiota. The beneficial effects of IMC are related to a reduction in TMAO; increases in genera *Acetatifactor*, *Bifidobacterium*, and *Eubacterium*; and decreases in genera *Phocacecola* and *Bacteroides*. Our findings afford insights into the targeting of the gut microbiota to deplete TMAO production, with therapeutic potential for the prevention of offspring hypertension programmed by maternal CKD, although these results still need further clinical translation.

## 1. Introduction

Up to 10% of the worldwide population is affected by chronic kidney disease (CKD) [1]. The prevalence of CKD among childbearing-aged women was estimated to be 3–4% [2]. In utero, environmental factors, such as maternal illness, determine the developmental programming of many diseases later in life, including hypertension and kidney disease [3,4]. This notion, preferentially termed “developmental programming”, forms the basis of the Developmental Origins of Health and Disease (DOHaD), demonstrating that fetus exposure to an adverse environment increases the risk of developing chronic diseases in adulthood [5].

While prior work indicated that the risks of adverse maternal and fetal outcomes are higher for women with CKD [6], little effort has been made to evaluate renal outcomes in adult offspring [7]. Using an adenine-induced CKD model, we formerly demonstrated that male rat offspring born under maternal CKD displayed hypertension and renal hypertrophy [8].

Trimethylamine-N-oxide (TMAO) is not only a gut microbial metabolite, but also a uremic toxin [9,10]. In the gut, bacteria degrade carnitine or choline to trimethylamine (TMA). TMA is converted into TMAO by flavin-containing monooxygenase (FMO). In CKD, the accumulation of TMAO correlates with cardiovascular mortality [10]. Additionally, TMAO has been connected to the development of hypertension [11,12]. Conversely, emerging evidence has shown the therapeutic potential of targeting the TMA-producing microbes and TMAO-related enzymes to protect against CKD and the associated comorbidity [13]. In a maternal high-fructose-diet model, perinatal 3,3-dimethyl-1-butanol (DMB, a choline analogue) treatment protected adult rat offspring against hypertension, which coincided with a reduction in TMAO formation [14].

Another choline analogue, iodomethylcholine (IMC), has also been utilized as a choline TMA lyase inhibitor to suppress TMAO production [15]. However, whether it can prevent maternal CKD-induced offspring hypertension is yet to be determined. To address the questions raised above, we examined whether maternal IMC treatment can protect adult offspring against maternal CKD-programmed hypertension and elucidate the underlying mechanisms in this study.

## 2. Results

### 2.1. Weight, BP, and Renal Function

We studied four groups of male offspring born to dams that received a normal diet chow (CN), an adenine diet (CKD), a normal diet chow and IMC administration (IMC), and an adenine diet and IMC administration (CKDIMC). Maternal IMC treatment caused a higher body weight (BW) in the IMC and CKDIMC groups compared to the CN and CKD groups (Table 1). The kidney weight (KW) was lower in the CN group compared to the other groups. Additionally, the CKD group exhibited a higher KW-to-BW ratio compared to the CN group.

**Figure 1:**
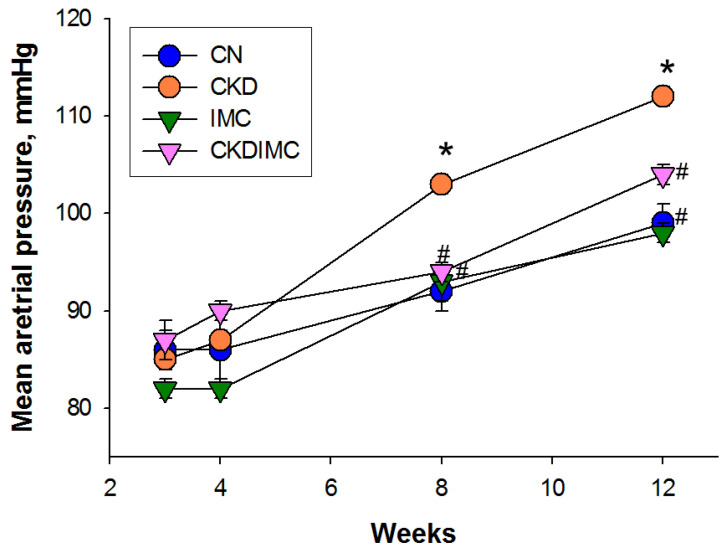
Effects of chronic kidney disease (CKD) and iodomethylcholine (IMC) on mean arterial pressure in rat offspring from 3 to 12 weeks of age. *N* = 8/group; * *p* < 0.05 vs. CN; # *p* < 0.05 vs. CKD.

Figure 1 shows that maternal CKD caused an elevation in mean arterial pressure (MAP) from 8 to 12 weeks (CKD vs. CN, *p* < 0.05). At weeks 8 and 12, CKD-induced increases in MAP were averted in the CKDIMC group (CKDIMC vs. CKD, *p* < 0.05). Table 1 illustrates that 12-week-old offspring in the CKD group had higher systolic and diastolic blood pressures, which IMC treatment prevented. The creatinine concentration in the plasma did not differ among the four groups.

Taken together, our data indicate that maternal CKD caused hypertension and renal hypertrophy in 12-week-old male progeny. CKD-induced offspring hypertension was improved by IMC treatment, while renal hypertrophy was not.

### 2.2. TMAO Metabolic Pathway

**Figure 2:**
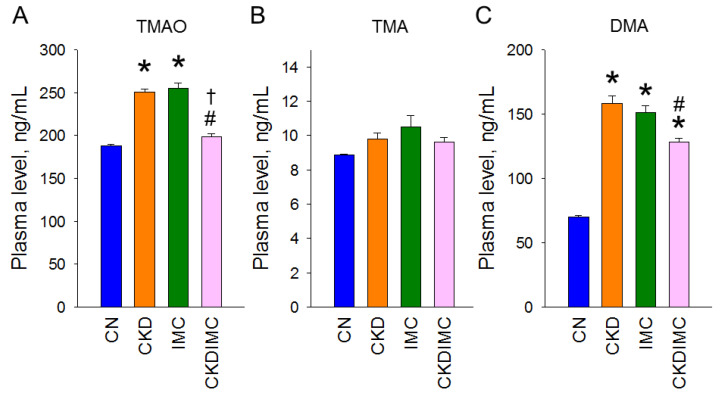
Effect of maternal chronic kidney disease (CKD) and iodomethylcholine (IMC) on plasma concentrations of (**A**) trimethylamine-N-oxide (TMAO), (**B**) trimethylamine (TMA), and (**C**) dimethylamine (DMA) in rat offspring at 12 weeks of age. *N* = 8/group; * *p* < 0.05 vs. CN; # *p* < 0.05 vs. CKD; † *p* < 0.05 vs. IMC.

To characterize the beneficial effects of IMC on CKD-induced hypertension, we determined the plasma concentrations of TMAO, TMA, and their metabolite dimethylamine (DMA). Figure 2A shows that plasma TMAO concentrations were higher in the CKD and IMC groups than in the CN group. The increase in TMAO in the CKD group was restored by IMC treatment (Figure 2A). No differences were found in plasma TMA levels (Figure 2B). Additionally, either CKD or IMC induced increases in DMA in the plasma (Figure 2C).

### 2.3. Oxidative Stress

**Figure 3:**
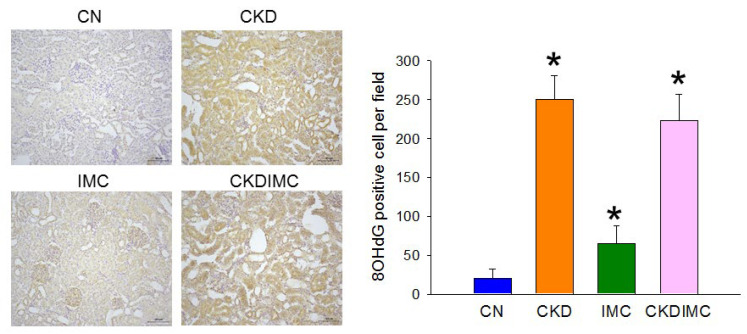
Light micrographs illustrating immunostaining for 8-OHdG in the offspring kidney (200×). * *p* < 0.05 vs. CN.

As oxidative stress represents a pathological link between TMAO and cardiovascular disease [16], we next determined whether the protective effects of IMC were related to reductions in oxidative stress. Immunohistochemical analysis employing 8-hydroxy-2′-deoxyguanosine (8-OHdG), a ubiquitous maker of oxidative DNA damage, was performed in offspring kidneys. As observed (Figure 3), immunostaining of 8-OHdG in glomeruli and tubular cells was intense in the CKD and CKDIMC groups compared with that in the CN group. IMC treatment also caused the slightly higher expression of 8-OHdG compared to the CN group.

### 2.4. Gut Microbiota Composition

**Figure 4:**
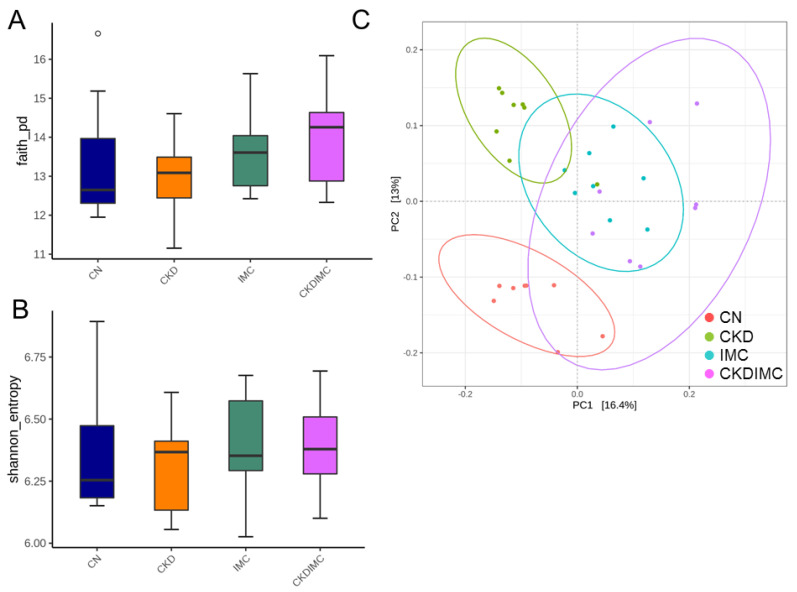
Bacterial α-diversity for gut microbial communities among four groups in (**A**) Faith’s phylogenetic diversity (PD) index and (**B**) Shannon index. The outlier is shown as a dot. (**C**) Bacterial β-diversity analysis using principal coordinate analysis (PCoA). Each dot represents the microbiota of a single sample, and the color of the dot reflects metadata for that sample.

Figure 4 illustrates the α- and β-diversity of bacterial communities. A comparison of α-diversity metrics showed no significant difference in the Faith phylogenetic diversity (PD) index (Figure 4A) and the Shannon index (Figure 4B). To assess the microbial community structural differences among the four groups, principal coordinate analysis (PCoA) based on the unweighted UniFrac metric was performed and showed that the microbial communities of the four groups were entirely separated into different clusters (Figure 4C). Moreover, the ANOSIM test yielded significant differences among the four groups (all *p* < 0.005).

**Figure 5:**
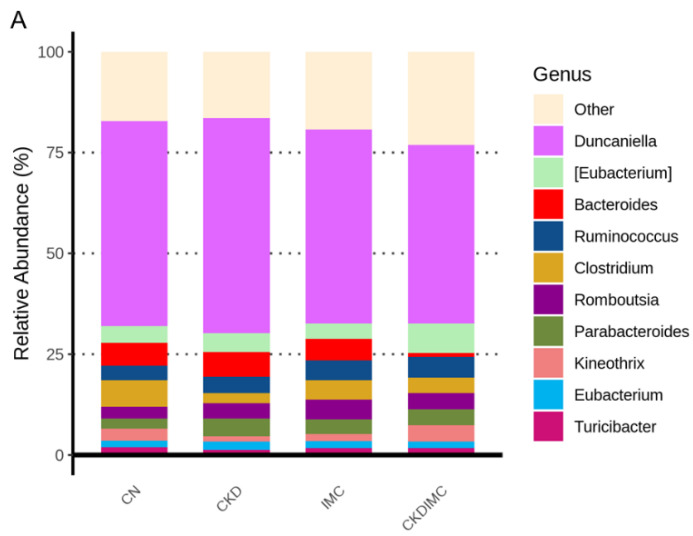

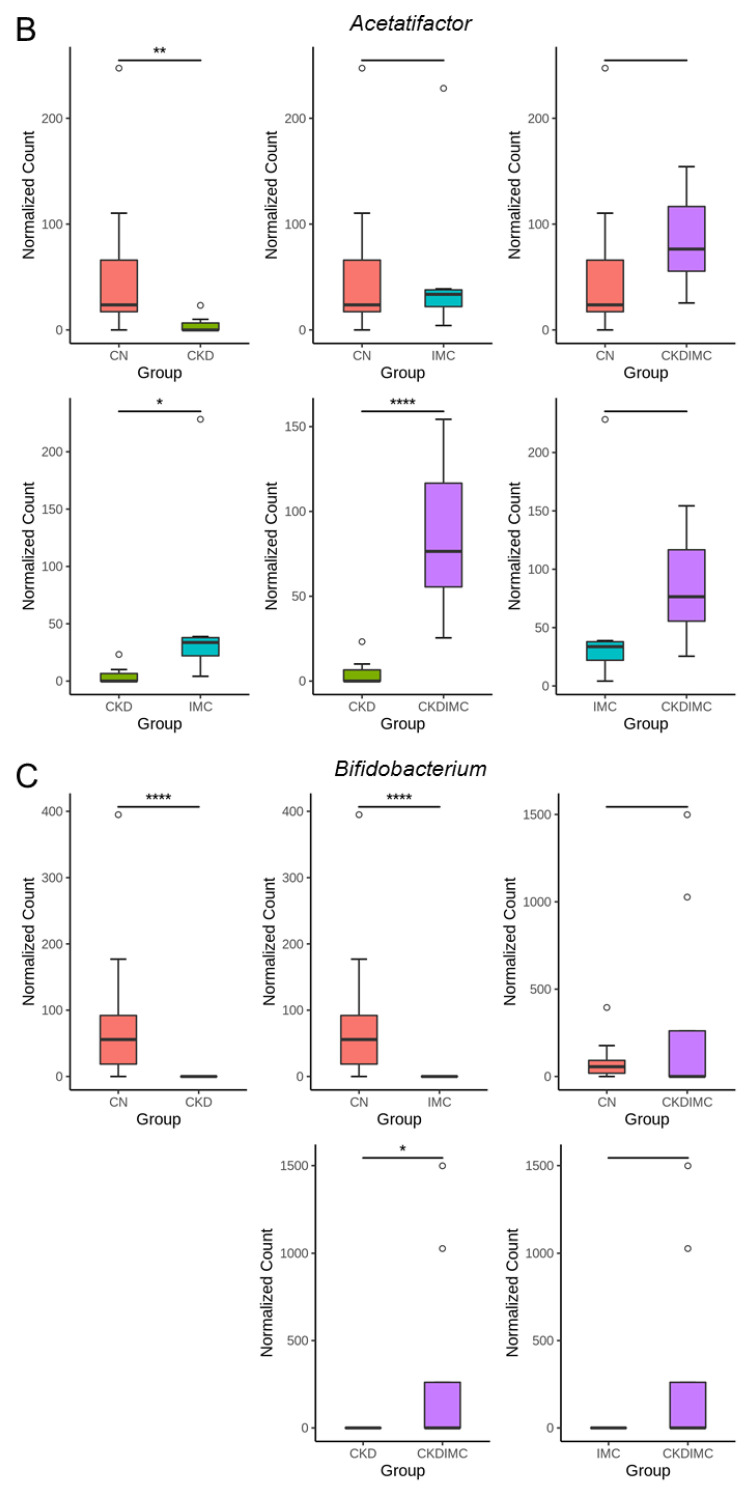

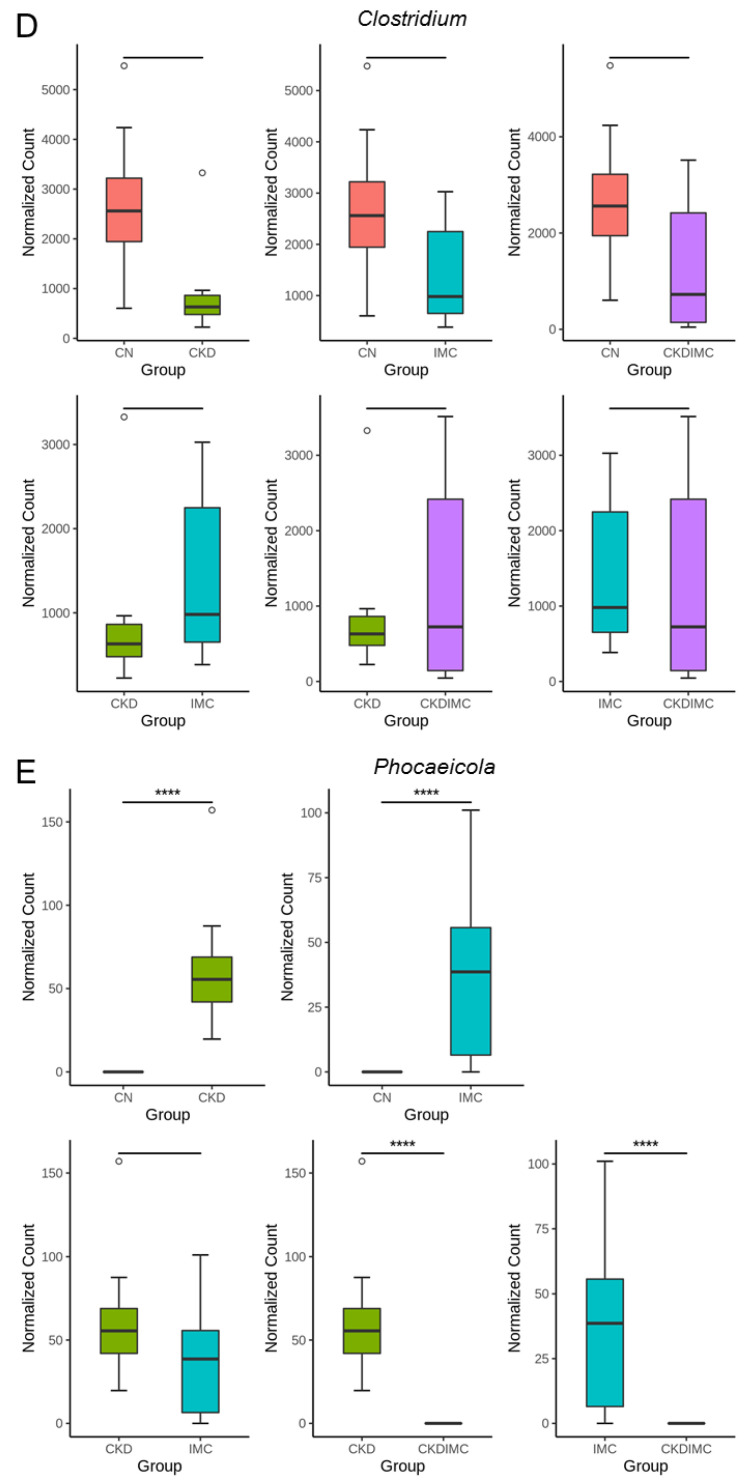
(**A**) Relative abundance of top 10 genera of the gut microbiota among the four groups. The comparison of genera (**B**) *Acetatifactor*, (**C**) *Bifidobacterium*, (**D**) *Clostridium*, and (**E**) *Phocaeicola* between the CN (orange) and CKD (green) groups (left panel), and between the CKD (green) and CKDIMC (purple) groups (right panel). The outliers are shown as dots. * *p* < 0.05; ** *p* < 0.01; **** *p* < 0.001.

Figure 5A demonstrates the major microorganisms’ genera found in offspring microbiota. Our data revealed that maternal CKD significantly reduced the abundance of genera *Acetatifactor*, *Bifidobacterium* and *Clostridium*, while it increased *Phocaeicola*, compared to the CN group (Figure 5B–E). Conversely, these alterations were restored by the IMC treatment.

**Figure 6:**
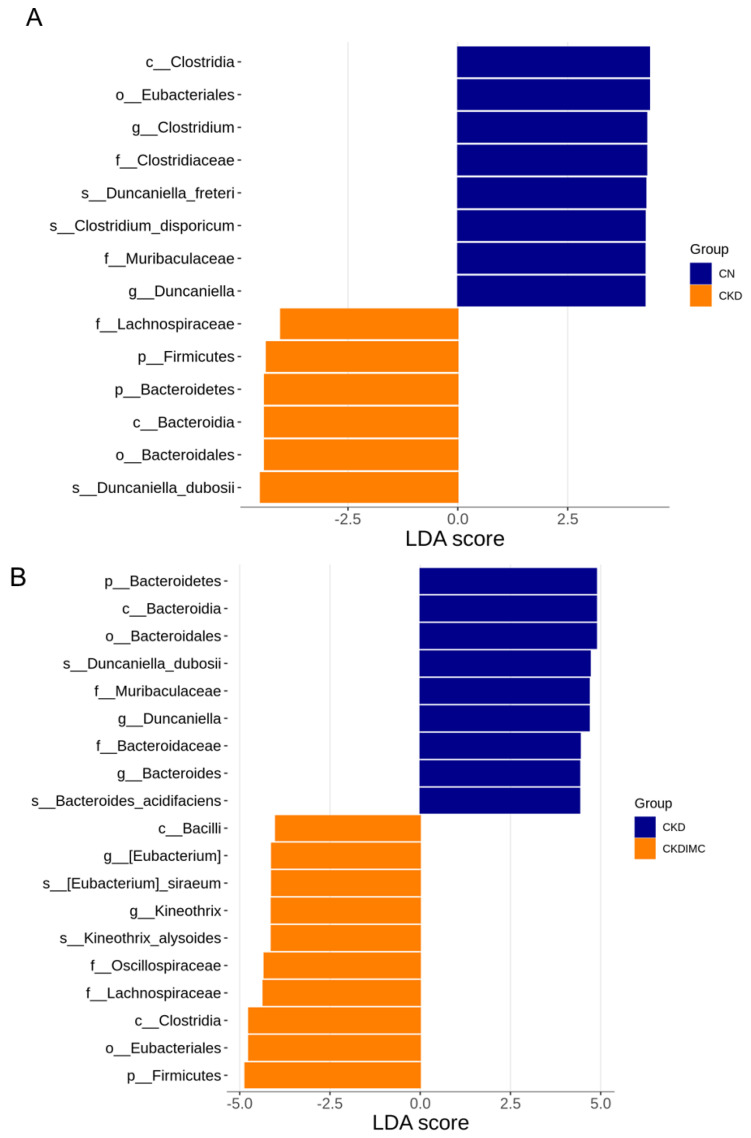
Linear discriminant analysis effect size (LEfSe) was assessed to identify most depleted and enriched bacterial taxa between (**A**) the CN and CKD groups, and (**B**) the CKD and CKDIMC groups. The linear discriminant analysis (LDA) score threshold was set to greater than 4.

The analysis of the linear discriminant analysis effect size (LEfSe) identified 14 taxa, with the greatest differences in abundance between the CKD and CN group (Figure 6A). Specifically, the genus *Duncaniella* and the family to which it belongs were more abundant in the CN group. Additionally, maternal CKD resulted in a decrease in the abundance of genus *Clostridium*. A total of 19 taxa exhibited significantly different abundances in the comparison between the CKD and CKDIMC groups (Figure 6B). Maternal IMC treatment caused the higher abundance of the genera *Eubacterium* and *Kineothrix* in the CKDIMC group compared to the CKD group, and a decrease in the genera *Bacteroides* and *Duncaniella*.

## 3. Discussion

This study affords insights into how the targeted inhibition of TMAO by IMC during pregnancy and lactation protects adult rat offspring against hypertension programmed by maternal CKD. Our most significant findings can be summarized as follows: (1) maternal CKD led to hypertension and renal hypertrophy in 12-week-old male offspring, and this was prevented by maternal IMC treatment; (2) maternal CKD-primed offspring hypertension was related to increases in plasma TMAO and DMA, oxidative stress, and alterations of the gut microbiota; (3) maternal CKD and IMC treatment caused the offspring gut microbiota profile to differentially change, leading to distinct enterotypes in each group; and (4) the beneficial effects of IMC involved a reduction in TMAO and DMA concentrations in the plasma; increases in genera *Acetatifactor*, *Bifidobacterium* and *Eubacterium;* and decreases in genera *Phocacecola* and *Bacteroides*.

In line with prior work showing that maternal CKD results in offspring with adverse renal outcomes [6,7,8], our results indicate that adult male offspring born to adenine-fed dams display hypertension and renal hypertrophy, an early feature of CKD. Although plasma creatinine concentrations remain not yet elevated, the accumulation of gut-microbiota-derived uremic toxin TMAO appeared. From our results, it is clear that the rise in BP occurs together with increases in plasma TMAO and DMA.

TMAO is regarded as a risk factor for cardiovascular mortality [9]. A previous meta-analysis reported that the TMAO concentration is dose-dependently associated with hypertension risk [17]. As circulating TMAO is cleared by the kidney, its accumulation in CKD is mainly due to reduced urinary clearance [18]. Of note is that results from this study demonstrate that maternal CKD-primed offspring hypertension is coincident with increases in plasma TMAO and its metabolite DMA in the absence of renal dysfunction. Potentially, elevations in plasma TMAO could be due to increased gut microbial TMA formation, subsequently enhancing the conversion of TMA to TMAO.

To the best of our knowledge, this is the first time that it has been demonstrated that maternal IMC treatment is beneficial for CKD-induced offspring hypertension, together with the reduction in TMAO formation. Several genera participating in TMA formation have been reported, including *Escherichia*, *Proteus*, *Anaerococcus*, *Edwardsiella, Providencia*, etc. [19]. However, IMC treatment had a negligible effect on their abundance. On the contrary, some gut microbes have the potential to convert TMAO back into TMA, such as *Bifidobacterium* [20]. We observed that maternal CKD decreased the abundance of *Bifidobacterium*, which IMC treatment prevented. Our results suggested that targeting depleting TMA levels using TMA-degrading bacteria might be a considerable approach in this regard.

Thus far, most studies have shown that the reduction in TMAO concentrations is achieved via TMA inhibitors [21]. One previous study demonstrated that oral administration of a TMA-metabolizing strain reduced the levels of TMA and TMAO and altered microbiota components [22]. On this point, our results revealed that IMC treatment increased the genus level of *Eubacterium* in the CKDIMC group, which could demethylate carnitine in an alternative pathway to reduce TMA [23]. Although maternal IMC treatment did not affect the diversity of the offspring gut microbiota in both the IMC and CKDIMC groups, its beneficial effect is related to certain microbes involved in TMA/TMAO metabolism.

In the present study, the beneficial microbes *Acetatifactor* and *Bifidobacterium* [24,25] were reduced in CKD offspring with hypertension. Nevertheless, maternal IMC treatment restored their abundance and reduced BP concurrently in the CKDIMC group. Our data are in accordance with former research showing that gut microbiota dysbiosis participates in hypertension and probiotics are beneficial in lowering BP. We also found that IMC treatment decreased the genus *Phocacecola*. It was recently shown that *Phocaeicola* might be related to preeclampsia occurrence [26], while currently there is a lack of information regarding its link with hypertension.

In addition, LEfSe analysis identified four bacterial taxa that have a strong relationship with hypertension, namely, the hypertension-depleted taxa *Eubacterium* and *Kineothrix* and the hypertension-enriched taxa *Bacteroides* and *Duncaniella*. Similar to our findings, prior work found that taxa enriched in hypertensive patients contain *Bacteroides* spp. [27]. Another study showed that hypertensive patients had a low abundance of *Eubacterium* [28]. Although the genus *Duncaniella* confers host protection from colitis [29], its role in hypertension remains largely unknown. Taken together, whether these specific taxa could serve as microbial markers for hypertension needs further in-depth research.

In support of oxidative stress having a pathological role in hypertension and kidney disease [3,30], maternal CKD-primed offspring hypertension was coincident with increased oxidative stress, represented by 8-OHdG staining. However, IMC treatment is able to reduce BP, but not oxidative stress, suggesting that the beneficial effects of IMC on BP might not be directly linked to oxidative stress.

Unexpectedly, maternal IMC treatment caused increased body weight in control offspring. Although prior work indicated that IMC treatment could attenuate high-fat-diet-induced obesity [31], its effect on body weight in normal controls remains unknown. Considering that IMC was not directly administered to offspring, its effect on body weight is presumably a programming effect rather than an acute effect. Our results suggest that maternal IMC treatment might have potentially adverse programming effects. Additional studies are needed to elucidate whether maternal IMC treatment might induce offspring obesity and to reveal the underling mechanisms. Moreover, maternal IMC treatment increased the plasma levels of TMAO and DMA in the controls. Although IMC had no significant effect on offspring BP, extensive research is needed to clarify whether maternal IMC intervention may result in any potential adverse long-term cardiovascular effects via mediating TMAO metabolism in adult offspring. Taken together, it would be logical to only apply maternal IMC treatment in the disease case, and it is not recommended for usual intake in healthy individuals.

Despite the relevant results presented here, our study still has some limitations. First, we did not examine female offspring, as hypertension occurred at an earlier age in males than females. Whether sex differences exist in response to IMC treatment awaits further clarification. Second, the gut microbiota profile was only examined in adult offspring, and not in dams or young offspring. Considering that programming effects vary during different developmental stages, it would be interesting to elucidate whether IMC treatment can regulate gut microbes involved in TMA/TMAO metabolism in both mother rats and neonate rats, and whether gut microbiota-derived TMAO is associated with offspring CKD later in life. Another limitation was that we did not study other organs involved in BP control. The protective effect of IMC on maternal CKD-induced offspring hypertension may be attributed to other organs.

## 4. Materials and Methods

### 4.1. Animal Protocol

This study was conducted in accordance with the guidelines of the Care and Use of Laboratory Animals of the National Institutes of Health. The experimental procedures were approved by the Institutional Animal Ethics Committee (permit number: 2021081203). A total of 12 virgin Sprague Dawley (SD) rats were randomly allocated to 4 groups (*n* = 3/group), namely, CN (normal chow diet), CKD (0.5% adenine diet), IMC (0.06% *w*/*w* IMC in chow, Sigma-Aldrich, Darmstadt, Germany), and CKDIMC (0.5% adenine diet plus IMC administration). To obtain a CKD model, eight-week-old female rats were fed a 0.5% adenine diet beginning 3 weeks before pregnancy and continuing throughout the pregnancy and lactation periods [8]. IMC was administered during gestation and lactation, and the dose used here was based on a previous report [13]. At 11 weeks of age, each female rat was mated with her assigned pair of males. All females successfully became pregnant, confirmed by the occurrence of a copulatory plug. As males have a higher likelihood of developing hypertension and display hypertension earlier than females [32], only male offspring were selected from each litter for the experiment. All litters were standardized to eight pups at one day of age. After weaning, all offspring were placed on a regular chow diet.

We used the tail-cuff technique (CODA system, Kent Scientific Corp., Torrington, CT, USA) to determine BP measurements in conscious retrained rats. One week ahead of the actual recording sessions, rats were placed in restraint holders and allowed to adapt to the procedures. At 12 weeks of age, all rat offspring belonging to the four experimental groups were sacrificed (*n* = 8/group). Prior to sacrifice, we collected fecal samples in the morning and stored them at −80 °C until analyses. Heparinized blood samples were obtained, aliquoted, and stored in freezers. Both kidneys were excised and weighed. One kidney was perfusion-fixed for immunohistochemical examination. Plasma creatinine concentrations were assessed by high-performance liquid chromatography (HPLC, HP series 1100; Agilent Technologies Inc., Wilmington, DE, USA).

### 4.2. Liquid Chromatography–Mass Spectrometry (LC–MS) Analysis

As previously described [8,14], plasma concentrations of TMA, TMAO, and DMA were determined by Liquid Chromatography–Mass Spectrometry (LC–MS) analysis. LC–MS analyses were carried out on an Agilent 6410 Series Triple Quadrupole MS (Agilent Technologies) coupled with an electrospray ionization source. Briefly, TMA, TMAO, and DMA were monitored in multiple-reaction-monitoring mode using characteristic precursor-product ion transitions: *m*/*z* 60.1→44.1, *m*/*z* 76.1→58.1, and *m*/*z* 46.1→30, respectively. For chromatographic separation, a SeQuant ZIC-HILIC column (150 × 2.1 mm, 5 μm; Merck KGaA, Darmstadt, Germany) was applied and protected by an Ascentis C18 column (2 cm × 4 mm, 5 μm; Merck KGaA, Darmstadt, Germany). Diethylamine was added to plasma samples as an internal standard. The mobile phase was a mixture (20:80, *v*/*v*) of methanol with 15 mmol/L ammonium formate (phase A) and acetonitrile (phase B) at a flow rate of 0.3–1 mL/min.

### 4.3. Metagenomics Analysis of Gut Microbiota

Fecal genomic DNA was extracted, as described previously [14]. Samples were analyzed by 16S rRNA metagenomics analysis at Biotools Co., Ltd. (New Taipei City, Taiwan). The entire 16S gene was amplified with barcoded multiplexed primers for PacBio SMRTbell library preparation (Menlo Park, CA, USA).

The high-throughput 16S rRNA sequencing data were analyzed via QIIME2 [33]. To construct amplicon sequence variants (ASVs), the DADA2 pipeline was carried out on QIIME2 [34]. To assess the phylogenetic distances between ASVs, they were aligned by MAFTT [35], and the phylogenetic tree was constructed with FastTree [36].

Two indices were used to measure α-diversity, namely Faith’s PD index and the Shannon index. To determine differences in microbiota communities across groups (i.e., β-diversity), we assess the ANOSIM and PCoA based on the unweighted UniFrac distance. Furthermore, LEfSe analysis was performed to determine the candidate taxa most likely to explain the differences between groups.

### 4.4. Immunohistochemistry Staining

We analyzed 8-OHdG expression in offspring kidneys, as it is a DNA oxidative damage marker [37]. Paraffin-embedded kidney sections at 3 μm thickness were deparaffinized with xylene and dehydrated with graded ethanol. After blocking with immunoblock (BIOTnA Biotech., Kaohsiung, Taiwan), the sections were incubated with 1:100 anti-8-OHdG antibody for 2 h (Catalog No.: N45.1, JaICA, Shizuoka, Japan). Immunoreactivity was detected using a polymer-horseradish peroxidase (HRP) labeling kit (BIOTnA Biotech.). We used 3,3′-diaminobenzidine (DAB) as the chromogen. A quantitative analysis of 8-OHdG-positive cells per microscopic field (×200) in the kidney section was performed as previously described [38].

### 4.5. Statistical Analysis

All data are presented as the mean ± standard error of means (SEM). Data were subjected to one-way analysis of variance (ANOVA) and multiple comparisons by using the post hoc Tukey test. A *p* < 0.05 was considered statistically significant. Statistical analysis was performed using SPSS version 17.0 (SPSS Inc., Chicago, IL, USA).

## 5. Conclusions

Besides a reduction in TMAO formation, maternal IMC treatment prevented maternal CKD-primed hypertension and renal hypertrophy in adult male offspring. This, alongside the alterations of the gut microbiota profile, indicates that targeting TMA-metabolizing bacteria to deplete TMA/TMAO production is a potential approach to protect adult offspring against maternal CKD-induced hypertension. Our study lays the foundation for further clinical translation of the therapeutic potential of TMA inhibitors in the prevention of maternal CKD-primed offspring hypertension and kidney disease.

## Figures and Tables

**Table 1:** Weight, BP, and renal function of male offspring at 12 weeks of age.

Group	CN	CKD	IMC	CKDIMC
Body weight (BW) (g)	304 ± 10	314 ± 12	355 ± 10 *,#	416 ± 16 *,#
Left kidney weight (g)	1.25 ± 0.04	1.45 ± 0.04 *	1.52 ± 0.05 *	1.78 ± 0.05 *
Left kidney weight/100g BW	0.41 ± 0.01	0.46 ± 0.01 *	0.43 ± 0.01	0.43 ± 0.02
Systolic blood pressure (mmHg)	128 ± 1	141 ± 1 *	126 ±1 #	135 ± 1 *
Diastolic blood pressure (mmHg)	85 ± 3	94 ± 2 *	85 ± 1 #	89 ± 2 #
Mean arterial pressure (mmHg)	99 ± 2	112 ± 1 *	98 ± 1 #	104 ± 1 #
Creatinine (μM/L)	14.5 ± 0.2	14.6 ± 0.1	15.3 ± 0.1	15.3 ± 0.2

*N* = 8/group; * *p* < 0.05 vs. CN; # *p* < 0.05 vs. CKD.

## Data Availability

Data are contained within the article.
